# Relationship between plasma homocysteine level and lipid profiles in a community-based Chinese population

**DOI:** 10.1186/s12944-017-0441-6

**Published:** 2017-03-14

**Authors:** Mohetaboer Momin, Jia Jia, Fangfang Fan, Jianping Li, Jingtao Dou, Dafang Chen, Yong Huo, Yan Zhang

**Affiliations:** 1 Department of Cardiology, Peking University First Hospital, Beijing, China; 2 Department of Epidemic & Biostatistics, School of Public Health, Peking University Health Science Center, Beijing, China; 3 Department of Endocrinology, Chinese People’s Liberation Army (PLA) General Hospital, Beijing, China

**Keywords:** Homocysteine, High-density lipoprotein cholesterol, Triglycerides

## Abstract

**Background:**

Previous studies established a possible link among hyperhomocysteinemia (HHcy), dyslipidemia, and atherosclerosis. However, there was limited epidemic data concerning the relation between HHcy and lipid profiles, especially in community-based Chinese populations. This study aim to investigate the association of plasma homocysteine (Hcy) level with lipid profiles in a Chinese community-based population without lipid-lowering treatment.

**Method:**

A total of 4660 Chinese subjects from a cohort of the Shijingshan district in Beijing were included in the analysis. Plasma total Hcy, serum lipid files including total cholesterol (TC), triglycerides (TG), low-density lipoprotein cholesterol (LDL-C), high-density lipoprotein cholesterol (HDL-C) as well as relevant metabolic risk factors were measured. Multivariate regression models adjusting for age, gender, smoking, drinking, physical activity, vitamin B supplement, body mass index, fasting blood glucose level, serum creatinine, systolic and diastolic blood pressure were used to evaluate associations of Hcy and lipid profiles.

**Result:**

Subjects were 56.75 ± 8.91 years old, and 38.15% were male. Median (IQR) Hcy was 11.98 (10.00–14.93) μmol/L, and 24.4% had HHcy (defined as Hcy ≥ 15 μmol/L). Mean (SD) baseline TC was 5.34 ± 0.98 mmol/L, LDL-C was 3.27 ± 0.81 mmol/L, and HDL-C was 1.43 ± 0.38 mmol/L. Median (IQR) of TG was 1.28 (0.91–1.85) mmol/L. In multivariable linear-regression analyses, lnHcy (ln transformation for Hcy) level was positively associated with lnTG (adjusted β = 0.075, SE = 0.021, *P* = 0.001). Using Hcy < 15 μmol/L as a reference, HHcy was independently associated with both lnTG (adjusted β = 0.056, SE = 0.020, *P* = 0.004) and lnHDL (adjusted β = −0.018, SE = 0.009, *P* = 0.038). In multivariable logistic-regression analyses, HHcy was associated with increasing risk of low HDL-C (HDL-C < 1.04 mmol/L; adjusted odds ratio [OR] =1.406, 95% confidence interval [CI]: 1.143 – 1.728, *P* = 0.001) and hypertriglyceridemia (TG ≥ 1.7 mmol/L; adjusted OR = 1.293, 95% CI: 1.096–1.524, *P* = 0.002) after adjusting the confounders. However, there were no significant associations between Hcy and TC or LDL-C.

**Conclusion:**

The present study showed that HHcy was independently associated with hypertriglyceridemia and low levels of HDL-C, which provides evidence that Hcy levels might affect HDL-C and TG metabolism.

## Background

Hyperhomocysteinemia (HHcy) has been regarded as a new modifiable risk factor for cardiovascular disease (CVD) through various mechanisms, including vascular endothelium damage, stimulation of smooth muscle cell proliferation, enhanced low-density lipoprotein cholesterol (LDL-C) peroxidation and thrombosis activation [1, 2]. Previous studies also established that there was a possible link among HHcy, dyslipidemia and atherosclerosis. Regarding Hcy, an inverse association between this amino acid and lipoproteins, especially high-density lipoprotein cholesterol (HDL-C), has been well described in humans and various animal models of HHcy [3]. HHcy might also increase the risk of CVD in dyslipidemia patients [4–6]. Although the mechanism of the link is not thoroughly known, recent studies strongly demonstrated the importance of the metabolic balance between S-adenosylmethionine (SAM), S-adenosylhomocysteine (SAH), phosphatidylcholine (PC), phosphatidylethanolamine (PE) and choline in Hcy metabolism, hypolipoproteinemia, liver function, and CVD [3, 7]. Several studies relating HHcy to disturbed HDL-C metabolism showed that Hcy can reduce circulating HDL-C via inhibiting ApoA-I protein synthesis and enhance HDL-C clearance [8, 9]. However, there are limited epidemic data about the relationship between HHcy and lipid profiles, especially in community-based Chinese populations. This study aims to investigate the association of plasma Hcy level with lipid profiles in a Chinese community-based population without lipid-lowering treatment.

## Methods

### Subject

Participants were from an atherosclerosis cohort survey performed in the Gucheng and Pingguoyuan communities of the Shijingshan district in Beijing, China from December 2011 to April 2012. The methods and primary results of this survey have been reported elsewhere [10]. A total of 4,660 eligible participants aged 40 years old and above were included in this analysis, after excluding those with missing covariates and those under lipid-lowering treatment. The proposal was approved by the ethics committee of both Peking University and Peking University First Hospital, and all subjects signed informed consent before enrollment.

### Data collection

Baseline data were collected by trained research staff according to a standard operating procedure. Each participant was interviewed using a standardized questionnaire designed specifically for the present study that provided information related to education status, medical history, body mass index (BMI), past and current medication use, and personal habits such as use of vitamin B (VB) supplements and exercise habits, as well as cigarette and alcohol consumption. Seated brachial blood pressure (BP) and pulse for each participant were obtained by trained researchers after subjects rested for 5 min; an Omron HEM-7117 electronic sphygmomanometer was used. The mean of three consecutive measurements was used in the analysis.

### Blood sample collection and laboratory methods

After an overnight fast of at least 12 h, a venous blood sample was obtained from the forearm of each participant. Plasma samples were separated within 30 min of collection and were stored at −80 °C. Plasma Hcy was measured using an electrochemiluminescence method at Southern Medical University Nanfang Hospital National Clinical Research Center for Kidney Disease in Guangzhou. Serum total cholesterol (TC), LDL-C, HDL-C, triglycerides (TG), fasting blood glucose (FBG), and creatinine (Scr) at baseline were measured on a Roche C8000 Automatic Analyzer in the laboratory of Chinese PLA General Hospital. According to the China Adult Dyslipidemia Prevention Guide (2007 Edition) criteria [11], we defined TC ≥5.18 mmol/L as hypercholesterolemia, LDL-C ≥3.37 mmol/L as a high level of LDL-C, HDL-C <1.04 mmol/L as low HDL-C, and TG ≥1.7 mmol/L as hypertriglyceridemia. According to results from previous studies [12], Hcy ≥ 15 μmol/L is often defined as HHcy. Additionally, in the present study, 15 μmol/L was the cut-off value of the upper quartiles of Hcy, so we defined Hcy ≥ 15 μmol/L as HHcy and divided the participants into two groups (Hcy ≥ 15 μmol/L and Hcy < 15 μmol/L) for further analysis.

### Statistical analysis

Categorical variables are expressed as numbers and percentages. Continuous variables are described using means with standard deviations for data with normal distribution, and medians with interquartile range (IQR) for non-normally distributed data. According to Hcy level, the participants were stratified into Hcy quartiles or two groups with cut-off value of 15 μmol/L. Univariate comparison was made between groups using the ANOVA test, and the *χ*2 test for categorical variables. The trend test (univariate analysis of each variate with Hcy quartiles) was used to test the linear trend of covariates with the increasing of Hcy. Multiple linear and logistic regression analysis adjusted for age, gender, smoking, alcohol drinking, VB supplement, BMI, FBG, pulse, physical activity, education level, BP and Scr was performed to assess the associations between each lipid profile and Hcy. All analyses were conducted using Empower(R) (www.empowerstats.com, X&Y solutions, inc. Boston MA). A *P*-value <0.05 was considered statistically significant.

## Results

Subjects were 56.75 ± 8.91 years old, and 38.15% were male. The median (IQR) value of Hcy was 11.98 (10.00 – 14.93) μmol/L, and 24.4% of subjects had HHcy. In order to have an even distribution in each group, the subjects were divided into subgroups using Hcy quartiles (Q1: 4.66–9.99 μmol/L; Q2: 10.00–11.97 μmol/L; Q3: 11.98–14.92 μmol/L; and Q4: 14.93–141.47 μmol/L). The trend test showed that with higher Hcy grade, subjects were older, more males, more smokers and drinkers. Also, levels of TG, Scr, BP, pulse, BMI and the proportion of physically active subjects were significantly higher. In contrast, with increased Hcy grade, HDL-C level, education level and the proportion of patients who took VB supplements were lower. No differences were observed for FBG. These data are presented in Table 1.

**Table 1:** Baseline Characteristics Stratefied by Hcy quartiles

Hcy (μmol/L)	Total	Q1	Q2	Q3	Q4	*P*-value	P for trend
Median (Min-Max)	11.98 (4.66–141.47)	8.83 (4.66–9.99)	11.02 (10.00–11.97)	13.23 (11.98–14.92)	18.50 (14.93–141.47)		
*N*	4660	1161	1169	1161	1169		
Age (year-old), mean ± SD	56.75 ± 8.91	53.31 ± 7.41	56.28 ± 7.97	58.59 ± 9.21	58.82 ± 9.72	<0.001	<0.001
Sex, *N* (%)							
Male	1778 (38.15%)	117 (10.08%)	319 (27.29%)	533 (45.91%)	809 (69.20%)	<0.001	<0.001
Female	2882 (61.85%)	1044 (89.92%)	850 (72.71%)	628 (54.09%)	360 (30.80%)		
Scr (μmol/L), mean ± SD	59.44 ± 14.23	57.22 ± 9.05	63.25 ± 11.66	69.08 ± 13.44	77.34 ± 17.78	<0.001	<0.001
TC (mmol/L), mean ± SD	5.34 ± 0.98	5.37 ± 0.99	5.40 ± 0.91	5.35 ± 0.97	5.23 ± 1.03	<0.001	<0.001
TG (mmol/L), Median (IQR)	1.28 (0.91–1.85)	1.24 (0.88–1.84)	1.25 (0.90–1.73)	1.28 (0.94–1.81)	1.35 (0.95–2.01)	<0.001	<0.001
HDL-C (mmol/L), mean ± SD	1.43 ± 0.38	1.49 ± 0.36	1.47 ± 0.36	1.43 ± 0.38	1.36 ± 0.39	<0.001	<0.001
LDL-C (mmol/L), mean ± SD	3.27 ± 0.81	3.28 ± 0.81	3.32 ± 0.77	3.30 ± 0.81	3.19 ± 0.85	<0.001	0.011
FBG (mmol/L), mean ± SD	6.16 ± 1.80	6.12 ± 1.80	6.20 ± 1.91	6.20 ± 1.85	6.10 ± 1.62	0.389	0.728
BMI (Kg/m^2^), mean ± SD	26.00 ± 3.37	25.86 ± 3.54	25.92 ± 3.31	26.07 ± 3.36	26.1 ± 3.28	0.130	0.019
SBP (mmHg), mean ± SD	133.89 ± 16.85	130.91 ± 16.34	132.90 ± 16.49	135.07 ± 16.91	136.65 ± 17.12	<0.001	<0.001
DBP (mmHg), mean ± SD	75.21 ± 9.95	74.48 ± 9.48	75.07 ± 9.85	74.85 ± 9.86	76.42 ± 10.47	<0.001	<0.001
Pulse (bpm), mean ± SD	78.51 ± 11.41	78.15 ± 10.36	78.25 ± 11.20	78.50 ± 11.61	79.13 ± 12.35	0.159	0.033
Education level, *N* (%)							
Elementary above	4581 (98.30%)	1150 (99.05%)	1153 (98.63%)	1137 (97.93%)	1141 (97.60%)	0.029	0.003
Illiterate	79 (1.70%)	11 (0.95%)	16 (1.37%)	24 (2.07%)	28 (2.40%)		
Smoking status, *N* (%)							
Current and former	1249 (26.80%)	96 (8.27%)	230 (19.67%)	356 (30.66%)	567 (48.50%)	<0.001	<0.001
Never	3411 (73.20%)	1065 (91.73%)	939 (80.33%)	805 (69.34%)	602 (51.50%)		
Alcohol drinking, *N* (%)							
Current and former	1204 (25.84%)	136 (11.71%)	242 (20.70%)	319 (27.48%)	507 (43.37%)	<0.001	<0.001
Never	3456 (74.16%)	1025 (88.29%)	927 (79.30%)	842 (72.52%)	662 (56.63%)		
Physical activity, *N* (%)							
Once a week above	3737 (80.43%)	896 (77.24%)	928 (79.66%)	971 (83.85%)	942 (80.79%)	<0.001	0.005
Others	912 (19.57%)	264 (22.76%)	237 (20.34%)	187 (16.15%)	224 (19.21%)		
Vitamin B supplement, *N* (%)							
Once a week above	386 (8.28%)	137 (11.80%)	105 (9.01%)	77 (6.64%)	67 (5.74%)	<0.001	<0.001
Others	4269 (91.72%)	1024 (88.20%)	1061 (90.99%)	1083 (93.36%)	1101 (94.26%)		

*Abbreviations*: *TC* Total cholesterol, *TG* Triglycerides, *HDL-C* High density lipoprotein cholesterol, *LDL-C* Low density lipoprotein cholesterol, *Hcy* Homocysteine, *Scr* Serum creatinine, *BMI* Body mass index, *FBG* Fasting blood glucose, *SBP* Systolic blood pressure, *DBP* Diastolic blood pressure, *IQR* Interquartile range

Multivariable regression analysis was carried out to assess whether Hcy was independently associated with these lipid profiles by adjusting for likely confounders. Model 1 was univariate analysis, Model 2 adjusted age and gender, model 3 adjusted age, gender, smoking, alcohol drinking, VB supplement, BMI, FBG, pulse, physical activity, education level, BP and Scr. In multivariable linear-regression analyses, lnHcy was positively associated with the lnTG in both models (adjusted β in full model = 0.075, SE = 0.021, *P* = 0.001). lnHcy was negatively associated with lnHDL (β = −0.024, SE = 0.010, *P* = 0.021) after adjustment for age and sex, but the association turned statistically insignificant after adjustment for all confounders (β = −0.016, SE = 0.010, *P* = 0.115). Though similar inverse trends were seen for lnTC and lnLDL, these were not statistically significant. When Hcy <15 μmol/L group was used as the reference, Hcy ≥ 15 μmol/L group was independently associated with lnTG (adjusted β in full model = 0.056, SE = 0.020, *P* = 0.004) and lnHDL (adjusted β in full model = −0.018, SE = 0.009, *P* = 0.038) as well. No positive associations were observed between HHcy and lnTC or lnLDL-C. These data are presented in Table 2.

**Table 2:** Multivariate linear regression for effects of Hcy on lipid profiles

	Model 1		Model 2		Model 3	
	β (SE)	*P*	β (SE)	*P*	β (SE)	*P*
LnTG (mmol/L)						
LnHcy	0.102 (0.020)	<0.001	0.077 (0.022)	<0.001	0.075 (0.021)	0.001
Hcy < 15 μmol/L	Ref.	-	Ref.	-	Ref.	-
Hcy ≥ 15 μmol/L	0.084 (0.018)	<0.001	0.061 (0.020)	0.001	0.056 (0.020)	0.004
LnHDL-C (mmol/L)						
LnHcy	−0.098 (0.010)	<0.001	−0.024 (0.010)	0.021	−0.016 (0.010)	0.115
Hcy < 15 μmol/L	Ref.	-	Ref.	-	Ref.	-
Hcy ≥ 15 μmol/L	−0.082 (0.009)	<0.001	−0.026 (0.010)	0.003	−0.018 (0.009)	0.038
LnTC (mmol/L)						
LnHcy	−0.033 (0.007)	<0.001	−0.002 (0.008)	0.739	−0.002 (0.008)	0.771
Hcy < 15 μmol/L	Ref.	-	Ref.	-	Ref.	-
Hcy ≥ 15 μmol/L	−0.029 (0.006)	<0.001	−0.005 (0.006)	0.486	−0.006 (0.007)	0.691
LnLDL-C (mmol/L)						
LnHcy	−0.044 (0.010)	<0.001	−0.016 (0.011)	0.095	−0.018 (0.012)	0.133
Hcy < 15 μmol/L	Ref.	-	Ref.	-	Ref.	-
Hcy ≥ 15 μmol/L	−0.039 (0.009)	<0.001	−0.017 (0.009)	0.080	−0.017 (0.010)	0.071

Model 1 univariate analysis

Model 2 adjusted for age and sex

Model 3 adjusted for age, sex, education level, physical activity, vitamin B supplement, body mass index, pulse, fasting glucose, smoking status, drinking status, systolic blood pressure, diastolic blood pressure, serum creatinine

*Abbreviations*: *TC* Total cholesterol, *TG* Triglycerides, *HDL-C* High density lipoprotein cholesterol, *LDL-C* Low density lipoprotein cholesterol, *Hcy* Homocysteine

Furthermore, HHcy was associated with increasing risk of low HDL-C (adjusted odds ratio [OR] in full model = 1.406, 95% confidence interval [CI]:1.143–1.728, *P* = 0.001) and hypertriglyceridemia (adjusted OR in full model = 1.293, 95% CI: 1.096–1.524, *P* = 0.002) in multivariable logistic-regression analyses. Similar significant findings were also found when analyzed the relation between InHcy and low HDL-C and hypertriglyceridemia. However, there were no significant correlations between HHcy and hypercholesterolemia or high LDL-C. These data are presented in Table 3.

**Table 3:** Multivariate Logistic Regression for Effects of Hcy on Dyslipidemia

	Model 1		Model 2		Model 3	
	OR (95% CI)	*P*	OR (95% CI)	*P*	OR (95% CI)	*P*
TG ≥ 1.70 (mmol/L)						
LnHcy	1.391 (1.192, 1.623)	<0.001	1.327 (1.117, 1.576)	0.001	1.267 (1.051, 1.525)	0.013
Hcy < 15 μmol/L	Ref.	-	Ref.	-	Ref.	-
Hcy ≥ 15 μmol/L	1.377 (1.195, 1.586)	<0.001	1.324 (1.136, 1.544)	<0.001	1.293 (1.096, 1.524)	0.002
HDL-C <1.04 (mmol/L)						
LnHcy	2.167 (1.797, 2.614)	<0.001	1.301 (1.049, 1.614)	0.017	1.277 (1.013, 1.613)	0.039
Hcy < 15 μmol/L	Ref.	-	Ref.	-	Ref.	-
Hcy ≥ 15 μmol/L	2.114 (1.769, 2.526)	<0.001	1.401 (1.154, 1.701)	<0.001	1.406 (1.143, 1.728)	0.001
TC ≥ 5.18 (mmol/L)						
LnHcy	0.746 (0.645, 0.864)	<0.001	0.991 (0.840, 1.168)	0.910	0.950 (0.798, 1.130)	0.562
Hcy < 15 μmol/L	Ref.	-	Ref.	-	Ref.	-
Hcy ≥ 15 μmol/L	0.754 (0.660, 0.862)	<0.001	0.930 (0.804, 1.075)	0.326	0.908 (0.779, 1.057)	0.214
LDL-C ≥ 3.37 (mmol/L)						
LnHcy	0.770 (0.662, 0.894)	<0.001	0.906 (0.766, 1.072)	0.250	0.904 (0.757, 1.080)	0.265
Hcy < 15 μmol/L	Ref.	-	Ref.	-	Ref.	-
Hcy ≥ 15 μmol/L	0.716 (0.624, 0.821)	<0.001	0.869 (0.750, 1.007)	0.061	0.910 (0.774, 1.071)	0.256

Model 1 univariate analysis

Model 2 adjusted for age and sex

Model 3 adjusted for age, sex, education level, physical activity, vitamin B supplement, body mass index, pulse, fasting glucose, smoking status, drinking status, systolic blood pressure, diastolic blood pressure, serum creatinine

*Abbreviations*: *TC* Total cholesterol, *TG* Triglycerides, *HDL-C* High density lipoprotein cholesterol, *LDL-C* Low density lipoprotein cholesterol, *Hcy* Homocysteine

## Discussion

The major findings of the present study are that HHcy status is independently associated with lower HDL-C and higher TG. The population of the present study was from a Chinese urban community. Compared to prior studies based on Chinese population, the lipid levels observed in this population are close to those reported in recent epidemic data [13–15]. The median level of Hcy was 11.98 μmol/L, which is comparable to other data [12].

The interaction between lipids and Hcy metabolism has been tested in several animal models for HHcy, hypercholesterolemia, or both [16–20]. There are also some invaluable clinical observations that demonstrate the possible link between Hcy and lipid metabolism pathways. Durdi et al. reported that in 126 myocardial infarction patients, Hcy was significantly and negatively correlated with HDL-C (*P* < 0.05, r = −0.93) and there was also a positive correlation between total Hcy and LDL-C (*P* < 0.05, r = 0.98) [21]. In 300 Indian subjects with proven coronary heart disease, Hcy was found to be positively associated with TG and VLDL-C, and negatively with HDL-C [22]. In 125 heterozygous familial hypercholesterolemia patients, Hcy (r = −0.370, *P* = 0.003) and methylenetetrahydrofolate reductase (MTHFR) TT genotype were associated with low HDL values [23]. Anan et al. reported similar results to those observed during the present study that Hcy is associated with TG and HDL-C, but not with TC nor LDL-C in 40 Japanese patients with diabetes [24]^,^ Rosa et al. reported that Hcy correlates negatively with ApoA-I and with HDL-C in elderly rural subjects from Sicily [25]. Focusing on Chinese subjects, in 2058 Chinese consecutive coronary artery angiographic patients, Hcy was found to be negatively correlated with HDL-C (r = −0.148, *P* < 0.001) [4]. In northern Chinese subjects, the prevalence of HHcy in the combined hyperlipidemia (high TG combined high TG) group has been reported to be significantly higher than that in the control with an OR of 3.339 [26]. However, not all prior studies have found correlations between HHcy and lipid profiles. Yadav reported that there was no significant correlation between plasma Hcy and TC, HDL-C, and TG in 60 ischemic heart disease patients [27]. A study that enrolled 155 diabetes patients and found no significant association between Hcy and lipids either [28]. Importantly, most recent data including 18297 US adults from the Vary Large Database of Lipids indicate that, in unadjusted analysis, levels of LDL-C, non-HDL-C and HDL-C were lower whereas levels of TG and VLDL-C were higher in the highest Hcy quartile, but after adjusting confounders the associations disappeared [29]. Compared to the results from the present study, the VLDL-21 study included a general population with similar age, sex ratio, and the Hcy level. However, the lipid levels were much lower than those observed in the present study. Also, information of lipid-lowering medication, one of the most confounders, was uncertain in VLDL-21 study. In a multiple linear regression model, only age, sex, HbA1c, insulin and Scr were adjusted. Other factors such as BMI, statin usuage, VB supplement, drinking status, and physical activities weren’t taken into account, which should partly explain the discrepancy between two studies.

In summary, while studies examining the association between HHcy and lipid profiles in humans have had mixed conclusions, the most consistent findings indicate that higher Hcy is associated with decreased serum HDL-C and increased TG, which are consistent with the results of the present study. Furthermore, we found that TC and LDL-C levels showed a downward trend in the HHcy group, but there is no significant difference. The clinical and epidemical data concerning correlations between Hcy and TC, LDL-C are very limited, indicating that more studies are needed. To the best of our knowledge, this was the largest study in Chinese population which could provide enough statistical power to evaluate the association of Hcy and lipid profiles. Besides, most prior researches were aimed at CVD patients or high risk patients, therefore, there is little reliable data about this phenomenon in community-based population. The population in the present study is community-based, which could provide more evidence for extrapolating the results to the general population. Additionally, most prior studies conducted univariate analysis or multivariate analysis adjusting for few confounders. Our study not only excluded subjects with lipid-lowering drugs which is one of the most important confounders, but also included many additional coviarates such as FBG, BMI, kidney function, physical activity, among others.

There are many experimental foundations that provide support for the hypothesis that Hcy affects lipid metabolism. (1) Prior study results suggest the mechanisms for HHcy are mainly related to the down regulation of key players in HDL production (Apo-AI, lecithin-cholesterol acyltransferase (LCAT)) [30] and the reducing of the liver Apo-AI mRNA expression [31]. (2) The inhibition of PC conversion to PE and the low ratio of PE/PC caused by HHcy are key issues in the relationship between HHcy and triglycerides accumulation [32, 33].(3) Hcy has been found to enhance the expression of sterol regulatory element-binding proteins (SREBPs) to increase intracellular accumulation of TC and TG [34]. (4) Hcy causes protein misfolding in the endoplasmic reticulum and oxidative stress, which might affect lipoprotein particle production [34–38]. (5) Global DNA hypomethylation has been suggested as a mechanism linking Hcy to lipid disorders and atherosclerosis in vascular smooth muscle cells [39]. (6) Plasma concentrations of TG have significantly important effects on the distribution of HDL subclasses, and HDL maturation might be abnormal and reverse cholesterol transport might be weakened in HTG patients [40]. Consequently, the findings in the present study might partially be explained by the interaction between TG and HDL-C.

The present study also has several limitations. It was a cross-sectional study, thus predictions about the incidence of dyslipidemia due to HHcy in the community-based population cannot be made from this study. Additionally, serum lipid profiles might relate to dietary habits that were not assessed in detail due to lack of such data. Further follow-up data in this cohort as well as independent replication are needed.

## Conclusion

The present study showed that HHcy status is independently associated with hypertriglyceridemia and low HDL-C levels, which provides evidence that Hcy might affect HDL-C and TG metabolism.
